# Supplementary material for: Investigating child sexual abuse material availability, searches, and users on the anonymous Tor network for a public health intervention strategy
Source: Sci Rep. 2024 Apr 3;14:7849. doi: 10.1038/s41598-024-58346-7 (PMC10991312; doi:10.1038/s41598-024-58346-7)
Supplement: Supplementary file 1 — Supplementary Information. [file 41598_2024_58346_MOESM1_ESM.pdf]

Investigating child sexual abuse material availability, searches, and users on the anonymous Tor network for a public health intervention strategy

Supplementary Information

Juha Nurmi[1,*], Arttu Paju[1], Billy Bob Brumley[2], Tegan Insoll[3], Anna K. Ovaska[3], Valeriia Soloveva[3], Nina Vaaranen-Valkonen[3], Mikko Aaltonen[4], and David Arroyo[5]

[1]Tampere University, Tampere, FI-33720, Finland
[2]Rochester Institute of Technology, Rochester, NY, 14623-5608, USA
[3]Suojellaan Lapsia, Protect Children ry., Helsinki, FI-00580, Finland
[4]University of Eastern Finland, Joensuu, FI-80101, Finland
[5]Consejo Superior de Investigaciones Científicas, Madrid, 28014, Spain
[*]juha.nurmi@tuni.fi

Contents

A Supplementary Methods **3**
- A .1 Phrase-based CSAM matching 3
- A .2 Naive Bayes classifier 4
- A .3 CSAM websites 6
  - A .3.1 Manual investigation 7
- A .4 Survey 8
  - A .4.1 Results on determinants of help-seeking behaviour 9
- A .5 Comparing age distributions statistically 12
  - A .5.1 Kolmogorov–Smirnov test 12
  - A .5.2 Chi-squared test 12
- A .6 Self-help services are reaching users 13
- A .7 6.7% of queries are CSAM-related 15
- A .8 Search behaviour is similar between years 15
- A .9 Compare to the IWF's Annual Report 2022 15

B Supplementary Tables **17**

C Supplementary Discussion **23**
C .1 Search chain analysis 23
C .2 Visitors and content volume for individual CSAM websites 23

D Supplementary Equations **25**

E Supplementary Notes **26**

A Supplementary Methods

A .1 Phrase-based CSAM matching

We randomly select 10,000 onion websites that were online in December 2022 and perform a basic keyword search (case-insensitive) on the titles of these websites (the HTML `title` element), containing the text that appears in the browser's title bar. This modest matching with 11 explicit CSAM phrases – including 'child porn', 'childxxx', 'lolita', 'preteen', and similar – produces 1,048 domains from 10,000 new onion domains. We read all of these titles, and they all made it clear that the website shares CSAM without false positive matches. Even with this simple, limited search, the results show that at least 10.5% of onion domains share CSAM.

Widening our search from the title element to the full text content, we find that 2,642 pages match our search terms.

Often, one website has multiple alternate onion domains; therefore, we wish to eliminate duplicates. We restrict our analysis to a single domain if multiple domains share identical content. We compare the title and sentences of the pages to detect duplicates.

We execute the search against the content of these distinct domains. There are duplicates because many websites offer at least five different domain addresses. In addition, there are empty websites without any content, and the Hello World default website on Apache or Nginx web servers.

As expected, the algorithm returns a smaller subset – 2,142 domains that present unique websites. The search returns 306/2,142 matches.

Manually reading the websites, we estimate the false positive (20 from 306, 6.5%) and false negative (6.0%) rates (see Supplementary Equations D). According to this keyword-based basic search with the stated false positive and false negative estimation, 18.5% of unique websites on Tor share CSAM.

In Figure 1 we present our search algorithm.

```python
# -*- coding: utf-8 -*-
''' Compare and search text content from the 2022-12 domain list '''
from difflib import SequenceMatcher # Module to compare sequences

def similar(txt1, txt2):
    ''' The title and first sentences used to estimate similarity '''
    return SequenceMatcher(None, txt1[0:200], txt2[0:200]).ratio() > 0.9

KEYWORDS = ["child porn", "childxxx", "lolita", "preteen", "...",
            "...", "...", "...", "...", "...", "..."]

content_list = []

# Check the domains from the random sample list of 10,000 onion domains
with open('10000_onions_2022-12-28.csv', 'r', encoding='utf-8') as onions:
    domain_list = [line.split(',')[0] for line in onions]
    for domain in domain_list: # Check each domain in the list
        # Saved HTML pages converted to text using 'import html2text'
        filename = './10000_pages/' + domain.split('.')[0] + '.txt'
        text = open(filename, 'r', encoding='utf-8').read().lower()
        findings = [word for word in KEYWORDS if word in text] # Search
        if any(True for txt in content_list if similar(txt, text)):
            continue # Similarity check returned True, skip
        print(f"{domain},{'/'.join(findings)}", flush=True)
        content_list.append(text) # This content printed once now
```

Figure 1: This is the search algorithm using CSAM-related vocabulary.

A .2 Naive Bayes classifier

To train classifiers, we manually produce representative CSAM (positive) and other (negative) website datasets. We achieve 93.8% accuracy with a basic naive Bayes classifier. Some legal adult pornography websites, like *PornHub*, provide an alternative onion domain accessible via Tor. Therefore, we include these in our training data to teach the classifier to differentiate between legal and illegal content. The classifier performs well and can even distinguish between legitimate pornography websites and unlawful CSAM websites.

Again, we read the textual content of these websites manually and compare the differences between these matching methods. Simple phrase matching and the classifier approaches have distinct differences: Simple term matching includes websites that are collecting links to other websites and have a collection of links with descriptions to CSAM websites, whereas the trained classifier does not match these websites as CSAM websites. Furthermore, the trained classifier understands to exclude websites that state they disallow the sharing of 'child porn'.

The *NLTK* Python library includes a decision tree classifier, which we train similarly. We output the decision tree structure for interpretation and compare the decision tree to the most informative features from the naive Bayes classifier.

The decision tree is overlearning extremely specialised text phrases but not

generic ones. The tree classification is also overly complicated. We are aware that decision trees are not robust and are susceptible to overfitting; we observe this with our training data since a slight change in the data results in a significant shift in accuracy. Instead, the naive Bayes classifier is able to generalise well from the training data.

In Figure 2, we present our Naive Bayes classifier for text-based CSAM detection.

```python
# -*- coding: utf-8 -*-
''' Naive Bayes classifier to detect CSAM websites '''
import re
from glob import glob
import nltk # pip install nltk==3.8.1

def features(filename):
    ''' Extract actual words, lower case, create all ngrams '''
    content = open(filename, 'r', encoding='utf-8').read().lower()
    text = re.sub(r'\W*\b\w{20,1000}\b|[^a-zA-Z0-9 ]', ' ', content)
    ngrams = list(nltk.everygrams(text.split(), max_len=3))
    return {' '.join(w): True for w in ngrams}

positive = [(features(f), 'CSAM') for f in glob('./data/csam/*.txt')]
negative = [(features(f), 'OTHER') for f in glob('./data/other/*.txt')]

threshold_p = int(0.8 * len(positive)) # 20/80 testing/training
threshold_n = int(0.8 * len(negative)) # 20/80 testing/training
train_set = positive[:threshold_p] + negative[:threshold_n]
test_set = positive[threshold_p:] + negative[threshold_n:]

print('Training: %d / Testing: %d', (len(train_set), len(test_set)))
classifier = nltk.classify.NaiveBayesClassifier.train(train_set)
print('Accuracy: ', nltk.classify.util.accuracy(classifier, test_set))
print(classifier.show_most_informative_features(50)) # child porn, ...
```

Figure 2: Naive Bayes classifier to detect CSAM-related vocabulary.

A .3 CSAM websites

This CSAM website in Figure 3 is easily detected as CSAM based on the text.

```
Little Porn Whore | Kink Child Porn # Little whore CP porn

  * Home
  * Login

400 GiB amateur child porn video

# 1985 - 2023 video and photo shooting

The latest update: Jan 2023

## The main cp child porn baby

The main section of our website with modern photos and videos CP

!child porn cp(/LittleWhores/child-porn-cp-1.jpg)
...
```

Figure 3: An illustration of the beginning text of a CSAM website.

This CSAM website in Figure 4 is available in several onion link directories categorised as 'child porn'.

```
iaudx324...kid.onion
Baby Love
Age: 0-3 years Age: 3-6 years Age: 7-13 years Age: 14-19 years All age
Baby Love # BABY LOVE

### Photo

### Video

# Age: 0-3 years

## Girls

## 56 GB photos and videos

![](http://baby-porn-gifs.iaudx324 ... kid.onion/images/102.jpg)
|  ![](http://baby-porn-gifs.iaudx324 ... kid.onion/images/92.jpg)
|  ![](http://baby-porn-gifs.iaudx324 ... kid.onion/images/61.jpg)
...
```

Figure 4: Some real CSAM websites, like *iaudx324 ... kid.onion*, do not use explicit sexual language, but notice the image file names.

A .3.1 Manual investigation

As a starting point to familiarise ourselves with the data, we randomly select the plain text representations of 1,000 onion websites that were online in January 2023 and have unique onion domain addresses. We read the text content of these websites to determine whether they share CSAM and what the English vocabulary is for this type of page. We learn that many websites have several alternative onion domains, so a unique onion domain does not reflect a unique website. Websites that share CSAM make this fact abundantly evident on the front page, as well as through the use of explicit, distinct wording. We have identified 221/1,000 domain addresses that share CSAM. We do not include borderline cases, but only websites that explicitly state they directly host underage sexual content, not other types of sexual content, or just mention sharing links to the content.

This human validation indicates that 22.1% of onion domains possess CSAM in January 2023.

We repeat this test by picking 1,000 onion domains from the year 2022 at random and examine the text on them. Again, we observe many borderline websites that describe the media on their pages like 'raping little girls films' or 'child love videos', but we refrain from classifying them as CSAM unless the ages of the victims or the fact that they are underage are indisputable. Eventually we find that 195 of these onion websites contain CSAM.

We repeated this test for the years 2018–2021 and see indications based on texts that the number of obvious CSAM websites is increasing. We also discover

that the phrases used to describe CSAM are evolving over time, meaning that our classifiers trained with the most recent data will fail to detect child sexual abuse terminology from the past. The more we investigate historical websites over time, the more we observe the specific terminology used in the past (like 'Pure Young Original', 'Best Onion C***d Porn Collection', 'BestCPPACK', and 'KidFlix'). This means that to maintain up-to-date vocabulary-based detection, constant retraining is required.

The results are similar to those of 2023 (22.1%, N = 221 of 1000), as this human validation indicates that 19.5% (N = 195 of 1000) of onion domains possess CSAM in 2022, 27.2% (N = 272 of 1000) in 2021, and 19.0% (190 of 1000) in 2020. While in 2019, 10.8% (N = 108 of 1000) and in 2018, 9.0% (N = 90 of 1000) of onion domains shared CSAM.

A .4 Survey

Three prominent Tor search engines display our questionnaire to the user who searched for CSAM. In this research, we analyse the responses of users who searched for CSAM on Tor web search engines using at least one of the 179 search phrases used to find CSAM. The search phrases in English, Russian, Spanish, and Swedish are only used to search for CSAM, e.g., the term 'childporn'. When a user makes a search containing any of these terms on one of the three Tor search engines, they are instead given the opportunity to voluntarily participate in the survey, which is available in 21 languages.

Our *Help us to help you* survey consists of 32 questions. We ask CSAM users about their thoughts, feelings, and actions related to their use of CSAM so that we can build a cognitive behavioural therapy-based anonymous rehabilitation programme for CSAM users. For this study, we analysed responses to 12 survey questions. All 12 questions are single-answer questions, i.e., the respondent is asked to pick one option from a predetermined list of answer options. As the answer options are somewhat ambiguous, respondents are asked to choose the option that best fits their experience. Some questions have the possibility of being explained further in an open-ended response. We analyse responses from all people who answered our *Help us to help you* survey from 5 May 2021 to 28 February 2023 (N = 11,470).

We analysed the data with both univariate and bivariate methods. The analyses in the main part of the text mainly describe the population seeking CSAM material on the Tor network, and these results provide a point of comparison to our other results. The bivariate analyses, on the other hand, deepen the picture of the factors associated with help-seeking for CSAM use. In these analyses, the outcome variable is based on help-seeking, whereas we selected the independent variables to measure both the intensity of CSAM use and the effects of CSAM use on the users themselves.

Independent variables:

Q1 : When I first saw CSAM/illegal violent material I was (age of onset)

Q2 : When I first saw CSAM/illegal violent material, it was (method of first exposure to CSAM)

Q3 : I have actively searched and viewed CSAM/illegal violent material for (duration of use)

Q4 : I search and view illegal violent material (frequency of use)

Q5 : I use images and videos (type of material)

Q7 : I would like to stop searching and viewing CSAM/illegal violent material (willingness to stop use)

Q8 : I have tried to stop searching and viewing CSAM/illegal violent material (tried to stop use)

Q13 : How are you feeling before you think, search or view CSAM/illegal violent material? (feelings before use)

Q20 : Have you had any self-harming thoughts or suicide attempts? (self-harm or suicide attempts)

Q22 : Have you experienced any difficulties related to your use of CSAM/illegal violent material? (experienced difficulties)

Q24 : Have you experienced difficulties in carrying out your ordinary daily routine and activities? (difficulties in daily life)

Q28 : Have you sought help to stop searching, using or sharing CSAM/illegal violent material? (help-seeking)

The survey asks respondents about their use of 'CSAM/illegal violent material'. In the study, we primarily refer to the results as relating to the use of CSAM, as we recruited the respondents based on their search for CSAM. We included the term 'illegal violent material' for those respondents who do not categorise the material they view as CSAM but indicated to us via their search terms that they are in fact searching for material depicting sexual abuse of children.

A .4.1 Results on determinants of help-seeking behaviour

Age of first exposure. The age of first exposure to CSAM has an impact on help-seeking rates and attitudes towards help-seeking. Respondents who were first exposed to CSAM between the ages of 14–17 are the most likely to report feeling afraid to seek help (25.6%, N = 523 of 2,043), and the least likely to have sought help (11.3%, N = 231 of 2,043). Respondents who were first exposed to CSAM over the age of 35 were the least likely to fear seeking help (13.7%, N = 74 of 540), and the most likely to have sought help (18.5%, N = 100 of 540). They were also most likely to have successfully received help (6.1%, N = 33 of

540). Simultaneously, this group is also the most likely to say that they have never sought help (55.4%, N = 299 of 540).

Type of material viewed, age of children depicted. Respondents who report viewing CSAM depicting infants and toddlers have a very high prevalence of help-seeking behaviour, with 27.3% (N = 100 of 367) having sought help. Those who search for violent or sadistic and brutal material are unlikely to seek help, with nearly three quarters reporting not having sought help (73.8%, N = 1,142 of 1,547). There is not a great difference in help-seeking based on the gender of the children depicted in the material viewed.

Duration and frequency of CSAM use. There is a strong association between rates of help-seeking and both the duration and frequency of use of CSAM. The longer the duration of use of CSAM, or the more frequent the use of CSAM, the more likely it is that the respondent has sought help.

Respondents who have viewed CSAM for less than a month are the least likely to have sought help: 58% (N = 1,666 of 2,866) report never seeking help, and only 11.3% (N = 325 of 2,866) report having sought help. Those who have viewed CSAM for over 5 years are the most likely to have sought help, with 18.8% (N = 249 of 1,323) reporting having sought help, and 6.1% (N = 81 of 1,323) having received help. The results indicate that individuals who have used CSAM for a longer period of time are more likely to recognise the need for help, and actively seek assistance to change their behaviour. This highlights the need to intervene with those who have been viewing CSAM for a shorter duration, in order to increase help-seeking at an earlier stage.

The frequency of CSAM use is also strongly associated with help-seeking. Respondents who view CSAM daily are the most likely to report having sought help (21.6%, N = 147 of 680), and the most likely to have successfully received help (7.6%, N = 52 of 680). This association may be explained in part by the detrimental impact that frequent use of CSAM may have on an individual's daily life, including impaired social and occupational functioning and deep distress, which may be a strong motivator to seek help to reduce use of CSAM in order to improve life situations.

In research on substance abuse, common reasons for help-seeking include having become a habitual user, taking a substance for a long time, and a need to take it daily. Such driving factors for help-seeking appear to be similar in this sample of CSAM users. This hypothesis is supported by the result that respondents who face more difficulties in carrying out ordinary daily routines and activities are more likely to have sought help to stop using CSAM. Those who experience such difficulties daily have one of the highest rates of help-seeking, with 30.8% (N = 176 of 571) having sought help. Respondents who experience some form of difficulty related to their use of CSAM – especially difficulties with substance abuse or mental health – are more likely to have sought help than those who experience no difficulties.

Depression, anxiety, self-harming thoughts, guilt and shame. Respondents who reported feeling depressed or anxious before using CSAM are more likely to have sought help for their use of CSAM than respondents with feelings such as optimism or sexual arousal. 20.5% (N = 183 of 893) have sought

help, and 4.8% (N = 43 of 893) have received help. Those with feelings of guilt and shame are also more likely to have sought help (16.6%, N = 188 of 1,129 have sought help). Respondents who feel optimistic and good about themselves before viewing CSAM are the least likely to have sought help. 63.6% (N = 1,230 of 1,935) report never seeking help, and only 11.1% (N = 214 of 1,935) report seeking help.

Thoughts of self-harm and suicide attempts are associated with a higher likelihood of seeking help. Respondents who reported never having experienced self-harming thoughts or suicide attempts had lower than average rates of help-seeking, with only 7.9% (N = 271 of 3,424) having sought help, whilst respondents who reported experiencing thoughts of self-harm 'all the time' have the highest help-seeking rates, with 30.2% (N = 153 of 506) having sought help. Those who reported having tried to self-harm or having attempted suicide were overall less likely to have sought help than those who report having very frequent self-harming thoughts, however, were more successful in receiving help, with 9.4% (N = 61 of 650) receiving help – over twice as likely as the average response of 3.7% (N = 257 of 7,013). Overall, CSAM users who experience mental health difficulties alongside or related to their use of CSAM are more likely to seek help to stop searching for a using CSAM.

A .5 Comparing age distributions statistically

The search data and the survey data provide age distributions for the children depicted in the CSAM. These distributions appear to be similar but with differences. We used the Kolmogorov–Smirnov test and the Chi-squared test to compare these distributions.

The resulting two-tailed p-values are close to zero, indicating that the survey responses and/or age-based searches are biased. The survey responses give the age ranges that respondents say they are interested in, whereas the search sessions reveal the precise age that people are most interested in.

In survey results, the distribution is smoother; respondents are interested in 11-year-olds and 12-year-olds with close frequency, and they are interested in 17-year-olds, too. In search sessions, however, CSAM users are not interested in 17-year-olds or 11-year-olds compared to seeking sexually explicit content from 12–16-year-olds, which is extremely popular. This may indicate that individuals who find 11- to 17-year-olds attractive continue to be particularly interested in 12- to 16-year-olds and search for content about them.

A .5.1 Kolmogorov–Smirnov test

In Figure 5, we use the Kolmogorov–Smirnov (KS) test. The KS test is a nonparametric test, without assumptions about the underlying distribution, and the KS test is designed to measure differences between distributions representing the maximum absolute difference between the two cumulative distributions.

We are defining and testing two hypothesis: the null hypothesis (H_0) that the two distributions are without significant differences, and the alternative hypothesis (H_a) that there are differences between the distributions.

We analyse the obtained two-tailed p-value to determine the similarity between the distributions. The p-value represents the probability that H_0 is true. A small p-value would suggest evidence against the H_0, indicating that the two distributions are significantly different. Our small p-value close to zero suggests that the observed differences between the distributions show biassed measurements and support the H_a.

A .5.2 Chi-squared test

In Figure 6, we conduct the same analysis using the Chi-squared test. The Chi-squared test is a nonparametric test, without assumptions about the underlying distribution. We obtain again a p-value close to zero, indicating that the survey responses and/or age-based searches are biased.

We conclude that the survey responses give the age ranges that respondents say they are interested in, whereas the search sessions reveal the precise age that people are most interested in.

```python
# -*- coding: utf-8 -*-
'''
Compare two age distributions using the Kolmogorov-Smirnov (KS) test
'''
import csv
from scipy.stats import ks_2samp # pip install scipy=1.10.1

# Load the data: Read the CSV files containing the distributions
data1 = [] # Ages 0-17
data2 = [] # Ages 0-17

with open('ages.csv', 'r', encoding='utf-8') as file1:
    reader = csv.reader(file1) # Ages from the search queries
    for row in reader:
        if len(row) == 3 and 'year' in row[0]:
            age = int(row[0].split('-')[0])
            if age < 18: # Do not include 18- and 19-year-olds
                data1 = data1 + [age]*int(row[1])

with open('survey_ages.csv', 'r', encoding='utf-8') as file2:
    reader = csv.reader(file2) # Ages from the survey
    for row in reader:
        if len(row) == 2 and 'year' in row[0]:
            age = int(row[0].split('-')[0])
            if age < 18:
                data2 = data2 + [age]*int(row[1])

# Perform the Kolmogorov-Smirnov test
print(ks_2samp(data1, data2).pvalue) # 5.36591e-34
```

Figure 5: Kolmogorov–Smirnov test to measure differences between distributions.

A .6 Self-help services are reaching users

When we study the searches, we discover that there are a few hundred queries from people who want to cease viewing CSAM and are concerned about their sexual interest in children, including queries 'overcome child porn addiction', 'how to stop watching child porn', 'child sexualization help program', 'help for child porn addiction', 'how to stop child porn addiction', 'self help child attraction', 'self help child porn addiction recovery', and 'self help program sexual attraction to kids'.

When a person searches for CSAM, three prominent Tor search engines provide only links to self-help programmes for those who are concerned about their interests, thoughts, feelings, or behaviours regarding children. Does this strategy work – do CSAM users visit and study these self-help programmes? Indeed, yes, as we illustrate next.

Ahmia.fi records which search result the user clicks to visit the website. This enables us to analyse which onion websites users visit, and compile a ranking of the most popular websites in 2023 (between 1 January 2023 and 26 April 2023,

```python
# -*- coding: utf-8 -*-
'''
Compare two age distributions using the Chi-Square test
https://docs.scipy.org/doc/scipy/reference/generated/scipy.stats.chisquare.html
'''
import csv
from scipy.stats import chisquare # pip install scipy=1.10.1
import numpy as np # pip install numpy=1.24.1

def chisquare_scale(n, y):
    '''
    Chi-Squared test with scaled inputs.
    This chi-squared test for goodness of fit requires the sums of both inputs
    the sums of both inputs to be (almost) the same, sum(data1) == sum(data2),
    hence, chisquare(data1, data2) would fail, and we need to scale the inputs.
    '''
    return chisquare(n, np.sum(np.array(n))/np.sum(np.array(y)) * np.array(y))

# Load the data: Read the CSV files containing the distributions
data1 = [] # Ages 0-17
data2 = [] # Ages 0-17

with open('ages.csv', 'r', encoding='utf-8') as file1:
    reader = csv.reader(file1) # Ages from the search queries
    for row in reader:
        if len(row) == 3 and 'year' in row[0]:
            if '18' in row[0] or '19' in row[0]:
                continue # Do not include 18- and 19-year-olds
            data1.append(int(row[1]))

with open('survey_ages.csv', 'r', encoding='utf-8') as file2:
    reader = csv.reader(file2) # Ages from the survey
    for row in reader:
        if len(row) == 2 and 'year' in row[0]:
            data2.append(int(row[1]))

print(chisquare_scale(data1, data2).pvalue) # 0.0
```

Figure 6: Chi-squared test to measure differences between distributions.

a total of 11,205,542 visits to 45,128 onion websites). Almost all of the top 200 onion websites are onion search engines, dark web marketplaces, and other sites that sell illegal goods, cryptocurrency services, and onion link directories. But there is one surprise among the top 10 most frequented websites: 'Sexual interest in children? – Help Page' ranks seventh[1] – with 17,794 visits.

For CSAM-related searches, only links to self-help programmes appear in the search results. According to search result click data, thousands of people visit these websites after searching for CSAM, and – according to one particular online self-help cognitive therapy website – these visitors stay on the site, browsing pages.

One of the self-help websites is maintained by Helsinki University Hospital

[1] http://helplinkshtttptrdoukunonaiansstlrx4yherxk6azymviqtgle2yd.onion/

(HUS) Psychiatry[2]. Between 30 June 2021 and 15 June 2022, the website was accessed 22,466 times. The programme contains information on how to take control of your emotions, thoughts, and behaviour in order to redirect the reader away from using CSAM. 1,422 of these visitors continue to explore the self-help programme from the portal page. They start by reading the first section on concerns about using CSAM. Those who start the self-help programme are very likely to continue: 97.1% (N = 1,381) continue to the second section on why some people use CSAM, and 73.1% (N = 1,040) continue to the third section to learn about the thought and behavioural patterns that guide actions and lead to CSAM use.

A .7 6.7% of queries are CSAM-related

We examine searches (N = 238,794,231, Ahmia.fi, February 2018 – February 2023) from users seeking content from the Tor network. Several of the top search phrases are obviously used to seek CSAM. We analyse these clear CSAM searches and discover that they remain popular throughout the years; for instance, 'child+porn' has been searched 1,216,983 times over the years, 'cp' 2,729,946 times, and 'pedo' 1,761,096 times.

We keep in mind that not all users actively seek CSAM, despite the fact that the majority of the most popular search phrases relate to sexual content. There are numerous additional suspicious searches, such as 'young+girls' and 'young+porn', in which a user may seek CSAM, but the aim is unclear. Thus, we do not consider these as explicit CSAM-related searches.

We discover that explicit CSAM-related search phrases account for 6.7% of the queries. Comparatively, we find that drugs and marketplace-related terminology make up only 1.4% of all searches. This indicates that a considerable proportion of Tor users seek CSAM. We combine the separate searches into a chain of searches to track user search sessions.

A .8 Search behaviour is similar between years

Before and after COVID-19 pandemic measures (lockdowns, individuals spending more time at home), there were no significant changes in the behaviour of CSAM users. We compare the CSAM search sessions from February 2019 to February 2020 (N = 2,245,445) to those from May 2020 to May 2021 (N = 2,442,588). The number of CSAM search sessions increases by 8.8% (N = 197,143), but the age distribution and number of specific searches remain similar.

A .9 Compare to the IWF's Annual Report 2022

Our age distributions from open-ended responses and search queries are consistent with the latest analysis of CSAM reports by the Internet Watch Foundation

[2] https://www.mielenterveystalo.fi/en/self-help/redirection-self-help-program-stop-using-csam

(IWF)[3], which shows that in 2022, 59.0% (149,401 of 253,387) of CSAM reports depicted children between 11 and 15 years old (the IWF's Annual Report 2022, Analysis by age).

The gender distribution is also relatively consistent with the latest IWF Annual Report 2022, which found 95.2% (204,989 of 215,387) of reported CSAM to depict girls (the IWF's Annual Report 2022, Analysis by sex). The analysis found that – although only 2.9% (6,253 of 215,387) of reported CSAM depicted boys – the severity of abuse was higher in material depicting boys (sic)[4].

---

[3] https://annualreport2022.iwf.org.uk/

[4] The IWF Annual Report 2022 visualises their raw data and displays their percentages in an imprecise manner (i.e., rounds 2.9% to 2%). We recalculated the percentages.

B Supplementary Tables

Question	Number of missing answers	Proportion missing answers (%)
Question 1	440	3.8
Question 2	1,871	16.3
Question 5	2,674	23.3
Question 7	2,904	25.3
Question 8	3,023	26.4
Question 13	3,654	31.9
Question 20	4,138	36.1
Question 22	4,368	38.1
Question 24	4,425	38.6
Question 28	4,457	38.9

Table 1: Help us help you survey – Missing information.

When I first saw CSAM/illegal violent material I was

	Freq	Valid (%)	Valid Cum. (%)	Total (%)	Total Cum. (%)
13 years old or under	4,048	36.70	36.70	35.29	35.29
14–17 years old	3,151	28.57	65.27	27.47	62.76
18–25 years old	2,149	19.48	84.75	18.74	81.50
26–35 years old	861	7.81	92.56	7.51	89.01
over 35 years old	821	7.44	100.00	7.16	96.16
NA	440			3.84	100.00
Total 11,470	100.00	100.00	100.00	100.00	

Table 2: Help us help you survey – Question 1 results

When I first saw CSAM/illegal violent material, it was

	Freq	Valid (%)	Valid Cum. (%)	Total (%)	Total Cum. (%)
Accidentally	4,843	50.45	50.45	42.22	42.22
Through my social connections	1,361	14.18	64.63	11.87	54.09
After searching actively for other hard-core material	1,527	15.91	80.54	13.31	67.40
After searching actively for sexual CSAM / illegal material	1,549	16.14	96.68	13.50	80.91
Other occasion, what?	319	3.32	100.00	2.78	83.69
NA	1,871			16.31	100.00
Total 11,470	100.00	100.00	100.00	100.00	

Table 3: Help us help you survey – Question 2 results

I have actively searched and viewed CSAM/illegal violent material for

	Freq	Valid (%)	Valid Cum. (%)	Total (%)	Total Cum. (%)
1–4 weeks	4,097	44.39	44.39	35.72	35.72
1–6 months	1,356	14.69	59.08	11.82	47.54
1 year	1,093	11.84	70.92	9.53	57.07
2 years	863	9.35	80.27	7.52	64.59
5 years or more	1,821	19.73	100.00	15.88	80.47
NA	2,240			19.53	100.00
Total	11,470	100.00	100.00	100.00	100.00

Table 4: Help us help you survey – Question 3 results

I search and view illegal violent material

	Freq	Valid (%)	Valid Cum. (%)	Total (%)	Total Cum. (%)
Occasionally	5,008	55.76	55.76	43.66	43.66
Once a month	1,206	13.43	69.18	10.51	54.18
Several times a month	1,070	11.91	81.10	9.33	63.50
Weekly	814	9.06	90.16	7.10	70.60
Daily	884	9.84	100.00	7.71	78.31
NA	2,488			21.69	100.00
Total	11,470	100.00	100.00	100.00	100.00

Table 5: Help us help you survey – Question 4 results

I use images and videos

	Freq	Valid (%)	Valid Cum. (%)	Total (%)	Total Cum. (%)
Related to violent or sadistic and brutal material	2,205	25.07	25.07	19.22	19.22
CSAM related to boys aged 4–13 years	1,617	18.38	43.45	14.10	33.32
CSAM related to girls aged 4–13 years	3,725	42.35	85.80	32.48	65.80
CSAM related to infants and toddlers aged 0–3 years	506	5.75	91.55	4.41	70.21
Other violent material, what?	743	8.45	100.00	6.48	76.69
NA	2,674			23.31	100.00
Total 11,470	100.00	100.00	100.00	100.00	

Table 6: Help us help you survey – Question 5 results

I would like to stop searching and viewing CSAM/illegal violent material

	Freq	Valid (%)	Valid Cum. (%)	Total (%)	Total Cum. (%)
Not at all	2,656	31.01	31.01	23.16	23.16
I have not thought about it	1,790	20.90	51.90	15.61	38.76
Weekly	1,071	12.50	64.41	9.34	48.10
Monthly	703	8.21	72.61	6.13	54.23
Nearly every time	2,346	27.39	100.00	20.45	74.68
NA	2,904			25.32	100.00
Total 11,470	100.00	100.00	100.00	100.00	

Table 7: Help us help you survey – Question 7 results

I have tried to stop searching and viewing CSAM/illegal violent material

	Freq	Valid (%)	Valid Cum. (%)	Total (%)	Total Cum. (%)
Not at all	3,247	38.44	38.44	28.31	28.31
Daily	1,136	13.45	51.89	9.90	38.21
Weekly	1,078	12.76	64.65	9.40	47.61
Monthly	1,000	11.84	76.49	8.72	56.33
Nearly every time	1,986	23.51	100.00	17.31	73.64
NA	3,023			26.36	100.00
Total 11,470	100.00	100.00	100.00	100.00	

Table 8: Help us help you survey – Question 8 results

How are you feeling before you think, search or view CSAM/illegal violent material?

	Freq	Valid (%)	Valid Cum. (%)	Total (%)	Total Cum. (%)
I feel optimistic and good about myself	2,321	29.70	29.70	20.24	20.24
I feel sexually aroused/agitated	2,852	36.49	66.18	24.86	45.10
I feel guilt and shame	1,299	16.62	82.80	11.33	56.43
I feel depressed or anxious	1,007	12.88	95.69	8.78	65.20
Other feelings, what?	337	4.31	100.00	2.94	68.14
NA	3,654			31.86	100.00
Total	11,470	100.00	100.00	100.00	100.00

Table 9: Help us help you survey – Question 13 results

Have you had any self-harming thoughts or suicide attempts?

	Freq	Valid (%)	Valid Cum. (%)	Total (%)	Total Cum. (%)
Never	3,647	49.74	49.74	31.80	31.80
Sometimes	1,482	20.21	69.95	12.92	44.72
Regularly	969	13.22	83.17	8.45	53.16
All the time	546	7.45	90.62	4.76	57.93
I have tried to harm/kill myself	688	9.38	100.00	6.00	63.92
NA	4,138			36.08	100.00
Total	11,470	100.00	100.00	100.00	100.00

Table 10: Help us help you survey – Question 20 results

Have you experienced any difficulties related to your use of CSAM/illegal violent material?

	Freq	Valid (%)	Valid Cum. (%)	Total (%)	Total Cum. (%)
Not at all	3,926	55.28	55.28	34.23	34.23
Yes, difficulties in close relationships	948	13.35	68.63	8.27	42.49
Yes, difficulties with substance abuse	798	11.24	79.86	6.96	49.45
Yes, difficulties with my mental health	1,228	17.29	97.16	10.71	60.16
Yes, other difficulties, what?	202	2.84	100.00	1.76	61.92
NA	4,368			38.08	100.00
Total	11,470	100.00	100.00	100.00	100.00

Table 11: Help us help you survey – Question 22 results

Have you experienced any difficulties related to your use of CSAM/illegal violent material?					
	**Freq**	**Valid (%)**	**Valid Cum. (%)**	**Total (%)**	**Total Cum. (%)**
Not at all	3,707	52.62	52.62	32.32	32.32
Yes, sometimes	1,438	20.41	73.03	12.54	44.86
Yes, monthly	760	10.79	83.82	6.63	51.48
Yes, weekly	559	7.93	91.75	4.87	56.36
Yes, daily	581	8.25	100.00	5.07	61.42
NA	4,425			38.58	100.00
Total	11,470	100.00	100.00	100.00	100.00

Table 12: Help us help you survey – Question 24 results

Have you sought help to stop searching, using or sharing CSAM/illegal violent material?					
	**Freq**	**Valid (%)**	**Valid Cum. (%)**	**Total (%)**	**Total Cum. (%)**
No never	3,441	49.07	49.07	30.00	30.00
I have thought about getting help	1,089	15.53	64.59	9.49	39.49
I am afraid to seek help	1,498	21.36	85.95	13.06	52.55
I have, but did not get any help	728	10.38	96.34	6.35	58.90
I have and I got help. What kind of help?	257	3.66	100.00	2.24	61.14
NA	4,457			38.86	100.00
Total 11,470	100.00	100.00	100.00	100.00	

Table 13: Help us help you survey – Question 28 results

Q1: When I first saw CSAM/illegal violent material I was
Q28: Have you sought help to stop searching, using or sharing CSAM/illegal violent material?

**Q1/Q28**	**No; never**	**No; thought about it**	**No; afraid of it**	**Yes; did not receive**	**Yes; received**	**Total**
**13 years old or under**	1,083 (47.3%)	396 (17.3%)	471 (20.6%)	248 (10.8%)	93 (4.1%)	2,291 (100.0%)
**14–17 years old**	987 (48.3%)	302 (14.8%)	523 (25.6%)	172 (8.4%)	59 (2.9%)	2,043 (100.0%)
**18–25 years old**	758 (51.4%)	219 (14.9%)	300 (20.4%)	148 (10.0%)	49 (3.3%)	1,474 (100.0%)
**26–35 years old**	264 (47.7%)	81 (14.6%)	109 (19.7%)	82 (14.8%)	18 (3.2%)	554 (100.0%)
**over 35 years old**	299 (55.4%)	67 (12.4%)	74 (13.7%)	67 (12.4%)	33 (6.1%)	540 (100.0%)
**NA**	50 (45.0%)	24 (21.6%)	21 (18.9%)	11 (9.9%)	5 (4.5%)	111 (100.0%)
**Total**	3,441 (49.1%)	1,089 (15.5%)	1,498 (21.4%)	728 (10.4%)	257 (3.7%)	7,013 (100.0%)

Table 14: Help us help you survey – Question 1 / 28 Crosstab results

Q2: When I first saw CSAM/illegal violent material, it was
Q28: Have you sought help to stop searching, using or sharing CSAM/illegal violent material?

**Q2/Q28**	**No; never**	**No; thought about it**	**No; afraid of it**	**Yes; did not receive**	**Yes; received**	**Total**
**Accidentally**	1,681 (52.0%)	482 (14.9%)	655 (20.3%)	325 (10.1%)	89 (2.8%)	3,232 (100.0%)
**Through my social connections**	433 (44.0%)	198 (20.1%)	214 (21.7%)	104 (10.6%)	35 (3.6%)	984 (100.0%)
**After searching actively for other hard-core material**	567 (47.3%)	203 (16.9%)	272 (22.7%)	113 (9.4%)	44 (3.7%)	1,199 (100.0%)
**After searching actively for sexual CSAM / illegal material**	588 (50.2%)	144 (12.3%)	259 (22.1%)	142 (12.1%)	39 (3.3%)	1,172 (100.0%)
**Other occasion, what?**	81 (39.9%)	21 (10.3%)	52 (25.6%)	16 (7.9%)	33 (16.3%)	203 (100.0%)
**NA**	91 (40.8%)	41 (18.4%)	46 (20.6%)	28 (12.6%)	17 (7.6%)	223 (100.0%)
**Total**	3,441 (49.1%)	1,089 (15.5%)	1,498 (21.4%)	728 (10.4%)	257 (3.7%)	7,013 (100.0%)

Table 15: Help us help you survey – Question 2 / 28 Crosstab results

Q3: I have actively searched and viewed CSAM/illegal violent material for
Q28: Have you sought help to stop searching, using or sharing CSAM/illegal violent material?

Q3/Q28	No; never	No; thought about it	No; afraid of it	Yes; did not receive	Yes; received	Total
**1–4 weeks**	1,666 (58.1%)	408 (14.2%)	467 (16.3%)	245 (8.5%)	80 (2.8%)	2,866 (100.0%)
**1–6 months**	393 (38.6%)	199 (19.5%)	285 (28.0%)	114 (11.2%)	28 (2.7%)	1,019 (100.0%)
**1 year**	363 (44.0%)	137 (16.6%)	201 (24.4%)	99 (12.0%)	25 (3.0%)	825 (100.0%)
**2 years**	276 (40.2%)	102 (14.8%)	203 (29.5%)	75 (10.9%)	31 (4.5%)	687 (100.0%)
**5 years or more**	595 (45.0%)	191 (14.4%)	288 (21.8%)	168 (12.7%)	81 (6.1%)	1,323 (100.0%)
**NA**	148 (50.5%)	52 (17.7%)	54 (18.4%)	27 (9.2%)	12 (4.1%)	293 (100.0%)
**Total**	3,441 (49.1%)	1,089 (15.5%)	1,498 (21.4%)	728 (10.4%)	257 (3.7%)	7,013 (100.0%)

Table 16: Help us help you survey – Question 3 / 28 Crosstab results

Q4: I search and view illegal violent material
Q28: Have you sought help to stop searching, using or sharing CSAM/illegal violent material?

Q4/Q28	No; never	No; thought about it	No; afraid of it	Yes; did not receive	Yes; received	Total
**Occasionally**	2,120 (58.2%)	479 (13.1%)	662 (18.2%)	289 (7.9%)	93 (2.6%)	3,643 (100.0%)
**Once a month**	330 (36.3%)	205 (22.6%)	239 (26.3%)	105 (11.6%)	29 (3.2%)	908 (100.0%)
**Several times a month**	303 (36.2%)	149 (17.8%)	251 (30.0%)	101 (12.1%)	32 (3.8%)	836 (100.0%)
**Weekly**	236 (37.3%)	105 (16.6%)	162 (25.6%)	103 (16.3%)	26 (4.1%)	632 (100.0%)
**Daily**	321 (47.2%)	99 (14.6%)	113 (16.6%)	95 (14.0%)	52 (7.6%)	680 (100.0%)
**NA**	131 (41.7%)	52 (16.6%)	71 (22.6%)	35 (11.1%)	25 (8.0%)	314 (100.0%)
**Total**	3,441 (49.1%)	1,089 (15.5%)	1,498 (21.4%)	728 (10.4%)	257 (3.7%)	7,013 (100.0%)

Table 17: Help us help you survey – Question 4/28 Crosstab results

Q13: How are you feeling before you think, search or view CSAM/illegal violent material?
Q28: Have you sought help to stop searching, using or sharing CSAM/illegal violent material?

Q13/Q28	No; never	No; thought about it	No; afraid of it	Yes; did not receive	Yes; received	Total
**I feel optimistic and good about myself**	1,230 (63.6%)	268 (13.9%)	223 (11.5%)	166 (8.6%)	48 (2.5%)	1,935 (100.0%)
**I feel sexually aroused / agitated**	1,267 (49.1%)	433 (16.8%)	584 (22.6%)	241 (9.3%)	55 (2.1%)	2,580 (100.0%)
**I feel guilt and shame**	388 (34.4%)	205 (18.2%)	348 (30.8%)	145 (12.8%)	43 (3.8%)	1,129 (100.0%)
**I feel depressed or anxious**	311 (34.8%)	131 (14.7%)	268 (30.0%)	140 (15.7%)	43 (4.8%)	893 (100.0%)
**Other feelings, what?**	149 (56.7%)	14 (5.3%)	31 (11.8%)	16 (6.1%)	53 (20.2%)	263 (100.0%)
**NA**	96 (45.1%)	38 (17.8%)	44 (20.7%)	20 (9.4%)	15 (7.0%)	213 (100.0%)
**Total**	3,441 (49.1%)	1,089 (15.5%)	1,498 (21.4%)	728 (10.4%)	257 (3.7%)	7,013 (100.0%)

Table 18: Help us help you survey – Question 13 / 28 Crosstab results

Q20: Have you had any self-harming thoughts or suicide attempts?
Q28: Have you sought help to stop searching, using or sharing CSAM/illegal violent material?

Q20/Q28	No; never	No; thought about it	No; afraid of it	Yes; did not receive	Yes; received	Total
**Never**	2,388 (69.7%)	361 (10.5%)	404 (11.8%)	193 (5.6%)	78 (2.3%)	3,424 (100.0%)
**Sometimes**	496 (35.4%)	341 (24.3%)	388 (27.7%)	137 (9.8%)	40 (2.9%)	1,402 (100.0%)
**Regularly**	190 (21.0%)	185 (20.4%)	336 (37.1%)	162 (17.9%)	32 (3.5%)	905 (100.0%)
**All the time**	103 (20.4%)	104 (20.6%)	146 (28.9%)	120 (23.7%)	33 (6.5%)	506 (100.0%)
**I have tried to harm / kill myself**	213 (32.8%)	80 (12.3%)	198 (30.5%)	98 (15.1%)	61 (9.4%)	650 (100.0%)
**NA**	51 (40.5%)	18 (14.3%)	26 (20.6%)	18 (14.3%)	13 (10.3%)	126 (100.0%)
**Total**	3,441 (49.1%)	1,089 (15.5%)	1,498 (21.4%)	728 (10.4%)	257 (3.7%)	7,013 (100.0%)

Table 19: Help us help you survey – Question 20 / 28 Crosstab results

Q22: Have you experienced any difficulties related to your use of CSAM/illegal violent material?
Q28: Have you sought help to stop searching, using or sharing CSAM/illegal violent material?

**Q22/Q28**	**No; never**	**No; thought about it**	**No; afraid of it**	**Yes; did not receive**	**Yes; received**	**Total**
**Not at all**	2,718 (71.5%)	368 (9.7%)	443 (11.7%)	211 (5.6%)	61 (1.6%)	3,801 (100.0%)
**Yes, difficulties in close relationships**	224 (24.7%)	292 (32.2%)	227 (25.1%)	131 (14.5%)	32 (3.5%)	906 (100.0%)
**Yes, difficulties with substance abuse**	117 (15.4%)	175 (23.0%)	284 (37.3%)	150 (19.7%)	35 (4.6%)	761 (100.0%)
**Yes, difficulties with my mental health**	278 (23.1%)	212 (17.6%)	474 (39.4%)	194 (16.1%)	46 (3.8%)	1,204 (100.0%)
**Yes, other difficulties, what?**	47 (24.6%)	14 (7.3%)	37 (19.4%)	17 (8.9%)	76 (39.8%)	191 (100.0%)
**NA**	57 (38.0%)	28 (18.7%)	33 (22.0%)	25 (16.7%)	7 (4.7%)	150 (100.0%)
**Total**	3,441 (49.1%)	1,089 (15.5%)	1,498 (21.4%)	728 (10.4%)	257 (3.7%)	7,013 (100.0%)

Table 20: Help us help you survey – Question 22 / 28 Crosstab results

Q24: Have you experienced any difficulties related to your use of CSAM/illegal violent material?
Q28: Have you sought help to stop searching, using or sharing CSAM/illegal violent material?

**Q24/Q28**	**No; never**	**No; thought about it**	**No; afraid of it**	**Yes; did not receive**	**Yes; received**	**Total**
**Not at all**	2,618 (72.5%)	337 (9.3%)	435 (12.0%)	169 (4.7%)	53 (1.5%)	3,612 (100.0%)
**Yes, sometimes**	429 (30.7%)	368 (26.3%)	391 (28.0%)	157 (11.2%)	52 (3.7%)	1,397 (100.0%)
**Yes, monthly**	101 (13.9%)	158 (21.7%)	306 (42.0%)	133 (18.3%)	30 (4.1%)	728 (100.0%)
**Yes, weekly**	90 (16.7%)	123 (22.9%)	160 (29.7%)	134 (24.9%)	31 (5.8%)	538 (100.0%)
**Yes, daily**	148 (25.9%)	75 (13.1%)	172 (30.1%)	96 (16.8%)	80 (14.0%)	571 (100.0%)
**NA**	55 (32.9%)	28 (16.8%)	34 (20.4%)	39 (23.4%)	11 (6.6%)	167 (100.0%)
**Total**	3,441 (49.1%)	1,089 (15.5%)	1,498 (21.4%)	728 (10.4%)	257 (3.7%)	7,013 (100.0%)

Table 21: Help us help you survey – Question 24 / 28 Crosstab results

C Supplementary Discussion

C .1 Search chain analysis

In Figure 7, we present an example of how we collected search session statistics.

```
# The CSAM_ss.txt file contains search chain lines with a timestamp, for example,
# one search session line is
#
# [1655760026, '16+years+old', '16+years+old+porn', 'cp+free', 'child+porn+free',
# 'teen+homemade', 'teen+homemade+free', 'teen+blowjob']
#
# We used the "grep" command for searching to collect statistics, for example, ages:
grep -ci "zero+year\|'0boy\|+0boy\|'0girl\|+0girl\|'0y\|+0y\|+0+y\|'0+y" CSAM_ss.txt
grep -ci "one+year\|'1boy\|+1boy\|'1girl\|+1girl\|'1y\|+1y\|+1+y\|'1+y" CSAM_ss.txt
grep -ci "two+year\|'2boy\|+2boy\|'2girl\|+2girl\|'2y\|+2y\|+2+y\|'2+y" CSAM_ss.txt
grep -ci "three+year\|'3boy\|+3boy\|'3girl\|+3girl\|'3y\|+3y\|+3+y\|'3+y" CSAM_ss.txt
grep -ci "four+year\|'4boy\|+4boy\|'4girl\|+4girl\|'4y\|+4y\|+4+y\|'4+y" CSAM_ss.txt
grep -ci "five+year\|'5boy\|+5boy\|'5girl\|+5girl\|'5y\|+5y\|+5+y\|'5+y" CSAM_ss.txt
grep -ci "six+year\|'6boy\|+6boy\|'6girl\|+6girl\|'6y\|+6y\|+6+y\|'6+y" CSAM_ss.txt
grep -ci "seven+year\|'7boy\|+7boy\|'7girl\|+7girl\|'7y\|+7y\|+7+y\|'7+y" CSAM_ss.txt
grep -ci "eight+year\|'8boy\|+8boy\|'8girl\|+8girl\|'8y\|+8y\|+8+y\|'8+y" CSAM_ss.txt
grep -ci "nine+year\|'9boy\|+9boy\|'9girl\|+9girl\|'9y\|+9y\|+9+y\|'9+y" CSAM_ss.txt
grep -ci "ten+year\|'10boy\|+10boy\|'10girl\|+10girl\|'10y\|+10y\|+10+y\|'10+y" CSAM_ss.txt
grep -ci "eleven+year\|'11boy\|+11boy\|'11girl\|+11girl\|'11y\|+11y\|+11+y\|'11+y" CSAM_ss.txt
grep -ci "twelve+year\|'12boy\|+12boy\|'12girl\|+12girl\|'12y\|+12y\|+12+y\|'12+y" CSAM_ss.txt
grep -ci "thirteen+year\|'13boy\|+13boy\|'13girl\|+13girl\|'13y\|+13y\|+13+y\|'13+y\|13teen" CSAM_ss.txt
grep -ci "fourteen+year\|'14boy\|+14boy\|'14girl\|+14girl\|'14y\|+14y\|+14+y\|'14+y\|14teen" CSAM_ss.txt
grep -ci "fifteen+year\|'15boy\|+15boy\|'15girl\|+15girl\|'15y\|+15y\|+15+y\|'15+y\|15teen" CSAM_ss.txt
grep -ci "sixteen+year\|'16boy\|+16boy\|'16girl\|+16girl\|'16y\|+16y\|+16+y\|'16+y\|16teen" CSAM_ss.txt
grep -ci "seventeen+year\|'17boy\|+17boy\|'17girl\|+17girl\|'17y\|+17y\|+17+y\|'17+y\|17teen" CSAM_ss.txt

grep -ci "eighteen+year\|'18boy\|+18boy\|'18girl\|+18girl\|'18y\|+18y\|+18+y\|'18+y\|18teen" ALL_ss.txt
grep -ci "nineteen+year\|'19boy\|+19boy\|'19girl\|+19girl\|'19y\|+19y\|+19+y\|'19+y\|19teen" ALL_ss.txt
```

Figure 7: Analysis of the user search chain, in this case for the ages mentioned in the search sessions.

Obviously, we attempt to capture all potential ages entered by users, but the method is always limited by the terms we choose. Furthermore, there may be false positive matches, despite the fact that we read through thousands of matches and did not observe these terms catching anything but references to ages.

C .2 Visitors and content volume for individual CSAM websites

According to Europol, technological advancements and the availability of CSAM content online have greatly increased its production, distribution, and use[5]. In 2020, the United Nations Office on Drugs and Crime (UNODC) reported that the magnitude and quantity of CSAM and distribution of the material on Tor make it exceptionally difficult to prosecute [6]. We list notable cases in which law enforcement were able to shut down and seize control of CSAM servers

[5] https://www.europol.europa.eu/cms/sites/default/files/documents/internet_organised_crime_threat_assessment_iocta_2020.pdf

[6] https://www.unodc.org/documents/southeastasiaandpacific//Publications/2021/Darknet_Cybercrime_Threats_to_Southeast_Asia_report.pdf

operating within the Tor network, revealing the number of users and material on the server.

Boystown (2021). In 2021, an onion website hosting CSAM with over 400,000 registered users was shut down as a result of an international investigation[7].

Welcome To Video (2018). This website operated from July 2015, distributing eight terabytes of CSAM and having 4,000 customers who paid with cryptocurrencies, until 5 March 2018, when the law enforcement operation seized the website and rescued at least 23 underage victims from the United States, Spain, and the United Kingdom who were being actively abused by site users[8]. The website contained more than two hundred thousand videos that had been downloaded collectively more than one million times[9].

Childs Play (2017). Before police took it down in 2017, the website attracted more than one million user registrations; between 3,000 and 4,000 of those users were active, and 100 were producers of CSAM videos[10][11].

The Giftbox Exchange (2016). In November 2016, law enforcement shut down this onion website, which had over 72,000 registered members and 56,000 postings. The website sorted CSAM by age ranges, including a sub-forum for 'Babies & Toddlers'[12].

Daisy's Destruction (2015). Peter Gerard Scully, an Australian citizen, was arrested in the Philippines in 2015 for sexually abusing multiple children, including raping and torturing babies, while operating a live-streaming service to broadcast the abuse on Tor for 10,000 US dollars per watch[13].

Playpen (2015). Before its closure in 2015, Playpen was one of the largest CSAM websites in the world[14], distributing tens of thousands of photos and videos to 215,000 registered users[15][16].

Lolita City (2013). When this website was shut down in 2013, it was one of the largest CSAM distributors on Tor, with over 100 gigabytes of media[17].

---

[7] https://www.vice.com/en/article/bvzxww/europol-took-down-dark-web-child-porn-site-boystown

[8] https://www.justice.gov/opa/pr/south-korean-national-and-hundreds-others-charged-worldwide-takedown-largest-darknet-child

[9] https://www.bbc.com/news/world-50073092

[10] https://www.theguardian.com/society/2017/oct/07/australian-police-sting-brings-down-paedophile-forum-on-dark-web

[11] https://www.vg.no/spesial/2017/undercover-darkweb/?lang=en

[12] https://www.justice.gov/usao-mdtn/pr/franklin-tennessee-man-and-three-others-sentenced-prison-engaging-global-child

[13] https://www.vice.com/en/article/59kye3/the-repulsive-world-of-hurtcore-the-worst-crimes-imaginable

[14] https://arstechnica.com/tech-policy/2017/05/creator-of-infamous-playpen-website-sentenced-to-30-years-in-prison/

[15] https://www.fbi.gov/news/stories/playpen-creator-sentenced-to-30-years

[16] https://www.justice.gov/opa/pr/kentucky-man-sentenced-prison-engaging-child-exploitation-enterprise

[17] https://web.archive.org/web/20211027153951/https://digital.library.unt.edu/ark:/67531/metadc700882/m1/1/high_res_d/R44101_2015Jul07.pdf

D Supplementary Equations

The basic CSAM-keyword search returns 306 matches from 2,142 unique websites. We estimate the accuracy of this matching method. We verify the rate at which the vocabulary-based search matches websites other than CSAM-websites and the extent to which it fails to detect apparent CSAM-websites. Therefore, we need to manually read through matched websites to estimate the false positive rate and manually read a sample of non-match websites to estimate the false negative rate.

After matching based on the CSAM-related vocabulary, we carry out manual validation to estimate the false positive and false negative rates. This method allows us to identify websites that state, for instance, 'child porn is not permitted' or use the phrase in a manner that indicates they do not share CSAM. Or indeed, share CSAM, but use rare vocabulary and avoid detection.

Manually, we find 20 false-positive websites; these are: (i) Forum discussions that do not involve child abuse but mention the terminology (seven in total). (ii) Child abuse is mentioned in the forum discussions, and links to the content are advertised (four in total). (iii) Link directories that declare they do not include webpages containing child abuse (three in total). (iv) Link directories that include webpages about child abuse and provide descriptions for them (two in total). (v) Marketplaces that explicitly prohibit the sale of 'child porn' (two in total). (vi) One coding website containing the line 'sudo cp /etc/fail2ban/jail.conf /etc/fail2ban/jail.local'. (vii) One webpage that contains random vocabulary.

We examine 100 domains that do not match our search criteria as a reference. We read the text content. Using this strategy, we find six websites that explicitly say they share CSAM media content. We can estimate the number of CSAM websites that we did not detect:

$$(2142 - 306) * 0.06 = 110$$

The false positive rate (FPR) is computed as FP / (FP+TN), where FP represents the number of false positives, TN represents the number of true negatives, and FP+TN is the total number of negatives.

$$FP + TN = 20 + (2,142 - 286 - 110) = 1,766$$
$$FPR = FP/(FP + TN) = 20/1,766 = 0.011325$$

We estimate the proportion of CSAM websites as follows:

$$matches - FP + ((samples - matches) * 0.06)/samples =$$

$$(306 - 20) + (2,142 - 306) * 0.06/2,142 = 0.184949$$

Based on estimates of false positives and false negatives, a keyword-based search suggests that 18.5 percent of unique websites on Tor share CSAM.

E Supplementary Notes

Associated publication is ‘Investigating child sexual abuse material availability, searches, and users on the anonymous Tor network for a public health intervention strategy’ in Scientific Reports, 2024.
